# Importance of the neutrophil-to-lymphocyte ratio as a marker for microbiological specimens in critically ill patients after liver or lung transplantation

**DOI:** 10.1007/s15010-024-02398-4

**Published:** 2024-11-25

**Authors:** Steffen B. Wiegand, Michael Paal, Jette Jung, Markus Guba, Christian M. Lange, Christian Schneider, Nikolaus Kneidinger, Sebastian Michel, Michael Irlbeck, Michael Zoller

**Affiliations:** 1 Department of Anaesthesiology and Intensive Care Medicine, Hannover Medical School, Carl-Neuberg-Str. 1, 30625 Hannover, Germany; 2 Institute of Laboratory Medicine, LMU University Hospital, LMU Munich, Munich, Germany; 3 Department of Medical Microbiology and Hospital Hygiene, Max-Von-Pettenkofer Institute, LMU Munich, Munich, Germany; 4 Department of General-, Visceral- and Transplant Surgery, LMU University Hospital Munich, Munich, Germany; 5 Department of Internal Medicine II, LMU University Hospital Munich, Munich, Germany; 6 Division of Thoracic Surgery, LMU University Hospital Munich, Munich, Germany; 7 Comprehensive Pneumology Center Munich, German Center for Lung Research (DZL), Munich, Germany; 8 Department of Medicine V, LMU University Hospital Munich, Munich, Germany; 9 Division of Pulmonology, Department of Internal Medicine, Medical University of Graz, Graz, Austria; 10 Department of Cardiac Surgery, LMU University Hospital Munich, Munich, Germany; 11 Department of Anaesthesiology, LMU University Hospital, Munich, Germany

**Keywords:** Neutrophil-to-lymphocyte ratio, IL-6, Intensive care medicine, Infection, Lung transplantation, Liver transplantation

## Abstract

**Purpose:**

The correct and early diagnosis of an infection is pivotal for patients, especially if the patients are immunocompromised. Various infection markers are used in clinics with different advantages and disadvantages. The neutrophil-to-lymphocyte ratio (NLR) is a cost effective parameter easily obtained without further investments. The aim of this study is to elucidate the value of the NLR in comparison to other established inflammation markers in patients in the intensive care unit who underwent liver or lung transplantation for the detection of bacterial and fungal specimens.

**Methods:**

In this retrospective single centre study infection marker and microbiology data of 543 intensive care cases of liver or lung transplanted patients in the intensive care unit after transplantation were analysed.

**Results:**

In total 5,072 lab work results and 1,104 positive microbiology results were analysed. Results of an area under curve analysis were better for the NLR (0.631; *p* < 0.001) than for CRP (0.522; *p* = 0.152) or IL-6 (0.579; *p* < 0.001). The NLR was independent of type of organ which was transplanted and gender of patients, whereas IL-6 values differed significantly between liver and lung transplanted patients and between male and female.

**Conclusion:**

All analysed inflammation markers are far from being perfect. The NLR is a sensitive marker with reasonable threshold for the detection of microbiological specimens independent of gender or type of organ transplanted. The use allows a more differentiated approach to face the challenge of bacteria and fungus in patients who underwent liver or lung transplantation.

**Graphical Abstract:**

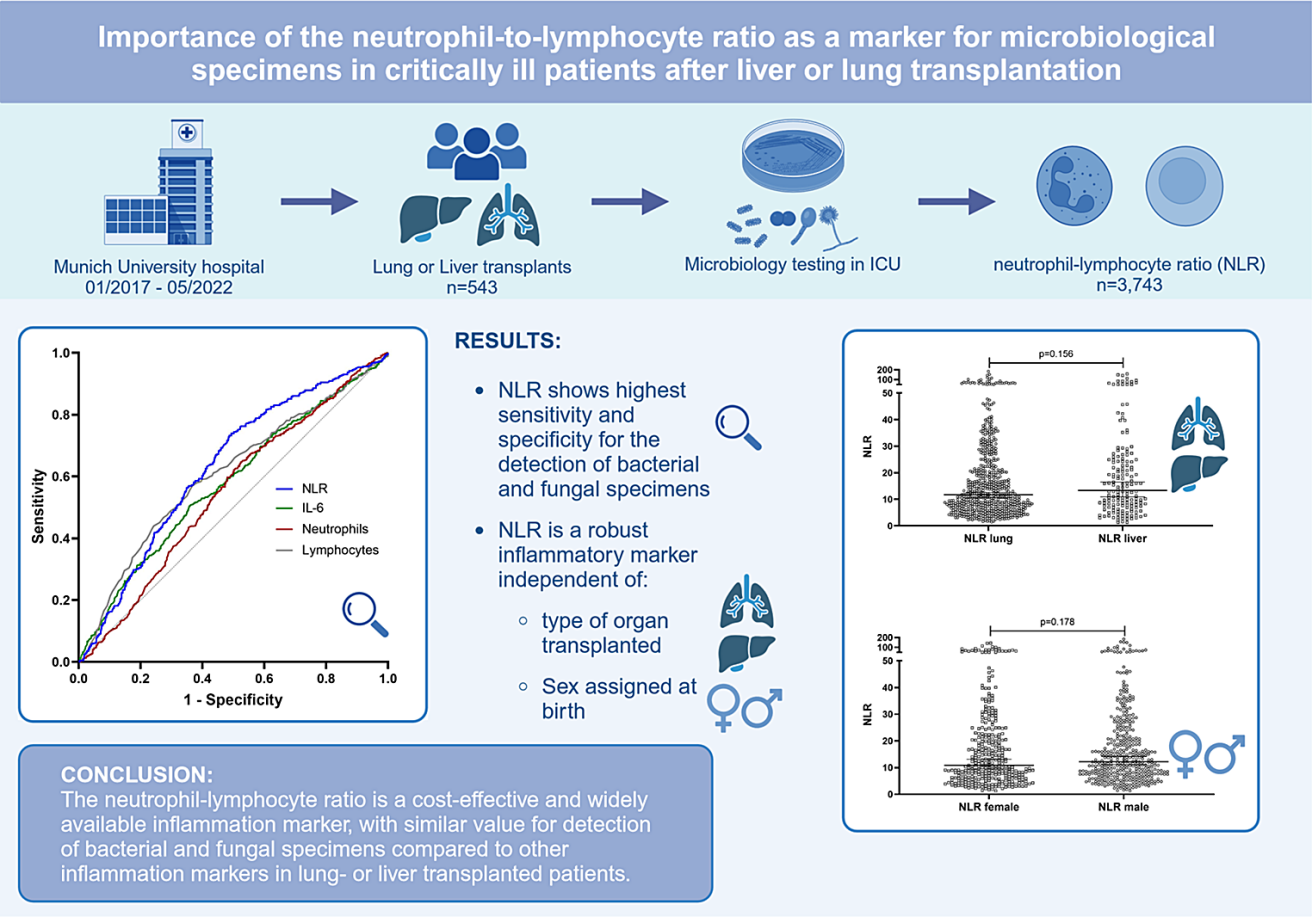

**Supplementary Information:**

The online version contains supplementary material available at 10.1007/s15010-024-02398-4.

## Introduction

The Surviving Sepsis Campaign emphasizes the pivotal role of early detection and treatment for sepsis; a delay will worsen the patients’ prognosis [1]. Initiating early goal-directed therapy is even more important for immunocompromised patients, such as solid-organ recipients [2]. Swift and accurate diagnosis of infections and sepsis remains challenging, although diagnostic measures to quantify inflammation biomarkers, such as C-reactive protein (CRP), white blood cell (WBC) count, serum procalcitonin (PCT), and interleukin-6 (IL-6), are widely available. While routine measurement of IL-6 has helped reduce the effective costs of sepsis treatment [3], the monetary costs for IL-6 quantification is a potential barrier in low- or medium-income countries. A refined evaluation of standard measures such as the neutrophil-to-lymphocyte ratio (NLR) could potentially help with the decision process without leading to immense cost.

The NLR is an indicator of an individual’s inflammatory status. The value of the NLR has been demonstrated in various medical scenarios, such as predicting mortality in significant cardiac incidents and trauma [4–6]. It can be a prognostic indicator during cancer therapy [7–10], and serves as a marker of inflammation, or infectious conditions [11–13]. In liver or lung transplanted patients, the NLR in serum or bronchoalveolar lavage is a prognostic marker for morbidity and mortality [14–18]. A differential blood count is required and the NLR is calculated by dividing absolute neutrophil count by absolute lymphocyte count.

An NLR reference range of 0.78–3.53 was established based on an evaluation of blood samples from healthy people taking part in a health care prevention program [19]. In patients with community-acquired bacterial infection with febrile illness, an NLR cutoff of 6.2 demonstrated a sensitivity of 91% and a specificity of 96% for detection of infection [20]. Higher NLR values (cutoff 9.03) were recorded for fungal infections [21].

The aim of this study was to evaluate the NLR as a marker for identifying bacterial and fungal samples in patients who have received lung or liver transplants.

## Patients and methods

### Ethical approval

This retrospective study was conducted in accordance with the guidelines of the Declaration of Helsinki, the principles of Good Clinical Practice and the standards of the local ethics committee. Data were analysed anonymously. The local ethical committee of Munich University Hospital at the Großhadern clinical site approved this retrospective, anonymous analysis of patient data (22–0287).

### Patients

In this retrospective, single centre study, postoperative cases of intensive care patients after lung or liver transplantation at Munich University Hospital, at the clinic site in Großhadern, Germany, were analysed. Lung and liver recipients who were admitted to the intensive care unit (ICU) between 01/2017 and 05/2022 were included.

The immunosuppression regimen for lung transplants included prednisolone, mychophenolate and tacrolimus, whereas liver transplants were treated with only prednisolone and tacrolimus. Patients generally received acyclovir for HSV prophylaxis. Lung transplant recipients also received antimycotic prophylaxis with posaconazole and TMP-SMX for Pneumocystis jirovecii prophylaxis. CMV prophylaxis was performed preemptively for all transplants.”

### Lab works

Routinely blood tests were conducted daily in the acute phase after liver or lung transplantation. The following parameters are included in this daily panel: IL-6, CRP, WBC, lymphocytes and neutrophils. PCT was not assessed routinely, but was assessed on demand. The timespan of the acute phase was dependent on the recovery pace of the patient and differed from patient to patient. Lab results of inflammation marker were matched as close as possible to the time-point of microbial testing. Only lab results within 48 h from the sampling date of positive microbial testing were used.

### NLR

The NLR is calculated by using the absolute (G/L) or relative (%) count of neutrophils and lymphocytes. The values obtained by absolute and relative count showed a very strong correlation (*r* = 0.997; *p* < 0.001) (Additional Fig. 1). Therefore we restricted further calculations to the NLR based on the absolute count.

**Fig. 1:**
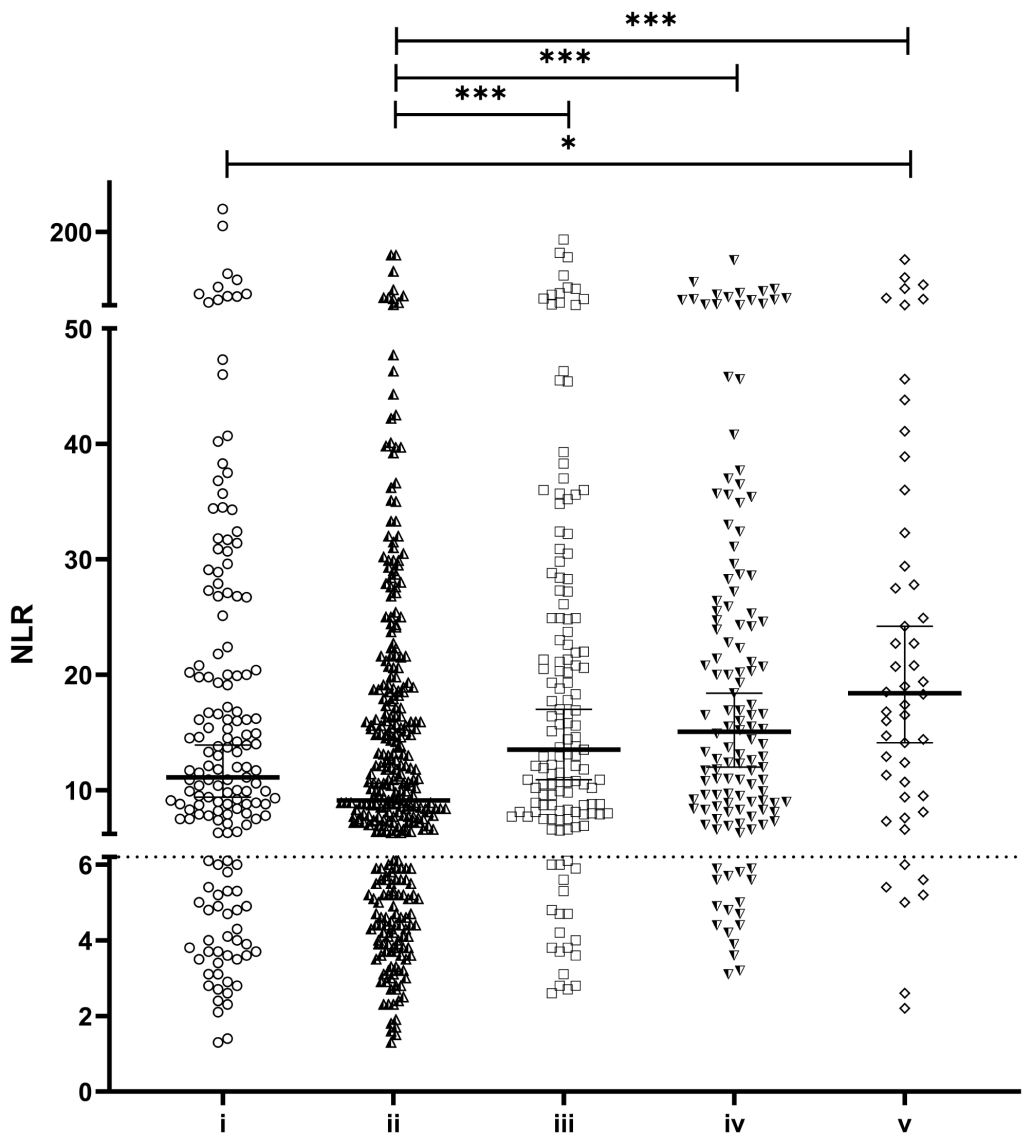
The NLR differs depending on the type of positive microbiology results. The NLR was highest in patients that were tested positive for the combination of Gram-positive and Gram-negative bacteria (v) (18.4; [14.1, 24.2]), but lowest in patients infected with Gram-negative bacteria (ii) (9.1; [8.5, 10.6]). The dotted line indicates the proposed threshold for NLR (6.2) for infection. (i: Gram-positive; ii: Gram-negative; iii: fungus; iv: combination of bacteria and fungus; v: combination of Gram-positive and Gram-negative)

### Microbial testing

Microbial testing for bacteria and fungi was performed routinely twice a week and additionally on demand using culture-based techniques. Sites for sampling included the airway tract (endotracheal suctioning, bronchoalveolar lavage), intrathoracic (chest drains), intra-abdominal (abdominal drains, bile drains, ascites), wounds and urinary tract. Positive bacteraemia results were not included in this analysis, as contamination could not be ruled out retrospectively. Positive screening results for perianal vancomycin-resistant enterococci, intranasal methicillin-resistant *Staphylococcus aureus* were considered to be colonization and were not included. To attenuate the issue of false positive microbiology results, we focused on patients who were tested positive multiple times for microbiology specimens.

### Statistical analysis

Statistical tests were performed using GraphPad Prism (v.8.4.3.; San Diego, CA, USA). Data are expressed as median values (95% confidence interval) unless otherwise specified. Normal distribution was tested with the Shapiro–Wilk test. The Mann–Whitney–U test with two-tailed p-value was used to compare two groups and the Kruskal–Wallis test with Dunn’s correction was used for multiple comparisons. Correlation was tested with the Spearman correlation test. The sensitivity of each biomarker was calculated in a contingency table. A receiver operating characteristic (ROC) curve was used to illustrate the performance of inflammation markers in detecting infection. The Youden index:

$$\:J=sensitivity+specificity-1$$

was calculated to find an optimal threshold for inflammation markers in Excel (v. 2311; Microsoft Corp., Redmond, WA, USA).

Values are presented as medians (95% confidence interval), unless otherwise stated. Other measures of central tendency and variability or reliability are available in the supplements (additional descriptive statistics.pdf). A p-value < 0.05 was considered significant (*p < 0.05; **p < 0.01; ***p < 0.001).

## Results

### Patients’ characteristics and labworks

In total, 543 cases of lung (*n* = 207) or liver (*n* = 336) recipients were analysed. The median age of the patients was 52 years and the majority were male (m: 326; f: 217). Of 5,072 lab works performed, marker availability sorted by frequency was as followed: leukocytes: 99.9%; neutrophils: 99.9%; CRP: 98.4%; IL-6: 95.7%; NLR: 73.8%, lymphocytes: 73.8%; and PCT 7.2% (Table 1).

**Table 1:** Baseline characteristics of patients and availability of inflammation marker. Baseline characteristics and inflammation markers in cases of lung or liver transplant patients in the ICU. References for markers are in […]

Lung or liver transplant cases in ICU, *n* (liver/lung)	207/336
Age, median years (SD)	52 (11.7)
Sex assigned at birth, n (F/M)	217/326
Lab works, n	5,072
IL-6, *n* = 4,854, 95.7% (median; 95% CI) [≤ 5.9 pg/mL]	30.5; 29.0–31.7
CRP, *n* = 4,989, 98.4% (median; 95% CI) [≤ 0.5 mg/dL]	4.1; 3.9–4.3
PCT, *n* = 364, 7.2% (median; 95% CI) [≤ 0.1 ng/mL]	1.8; 1.4–2.4
Leukocytes, *n* = 5,067, 99,9% (median; 95% CI) [3.9–9.8 G/L]	10.2; 9.95–10.4
Neutrophils, *n* = 5,067, 99.9% (median; 95% CI) [1.78–6.23 G/L]	8.11; 7.94–8.29
Lymphocytes, *n* = 3,743, 73.8% (median; 95% CI) [1.05–3.24 G/L]	0.58; 0.56–0.6
NLR, *n* = 3,743, 73.8% (median; 95% CI) [0.78–3.53] [19]	13.8; 13.3–14.3

### The NLR is increased in patients with positive bacterial and fungal samples

To evaluate the discriminating ability of the NLR, inflammation markers were analysed in patients who underwent liver or lung transplantation with multiple time positive microbial testing. Depending on the kind of microbe, different values for the NLR were obtained. The highest values were found in patients with positive test results for the combination of Gram-positive and Gram-negative bacteria (v) (18.4 [14.1, 24.2]), followed by patients with bacteria and fungi (iv) (15.1 [12.0, 18.4] (Fig. 1). In patients positive for only fungi (iii), the NLR was 13.5 [10.9, 17.0], and in patients with only Gram-positive bacteria (i) the NLR was 11.1 [9.4, 13.9]. The lowest NLR was found in patients with Gram-negative bacteria only (ii) (9.1) [8.5, 10.6].

A NLR value of 6.2 (9.03), was proposed as a threshold with both sensitivity and specificity > 90% for bacterial infection (fungal infection) [20, 21]. The frequency of NLR > 6.2 was the highest in patients tested positive for the combination of bacteria and fungi (iv) (87.1%), followed by the combination of Gram-positive and Gram-negative bacteria (v) (86.0%). In patients positive for fungi alone (iii), NLR > 6.2 was 86.0%, for Gram-positive bacteria alone (i) 74.8% and Gram-negative bacteria alone (ii) 70.8%.

### The NLR shows higher sensitivity and specificity than other inflammatory markers

**Fig. 2:**
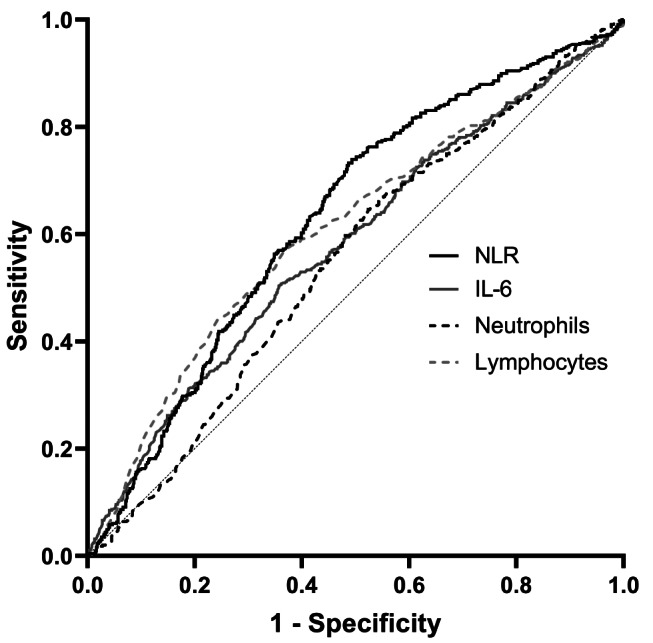
The NLR showed the highest sensitivity and specificity of inflammation markers in patients with multiple times positive microbial results. NLR showed highest sensitivity to specificity relationship in ROC analysis (AUC: 0.632; *p* < 0.001. The AUC for IL-6 was smaller (AUC: 0.580; *p* < 0.001). AUC of neutrophils and lymphocytes were 0.550 (*p* = 0.001) and 0.608 (*p* < 0.001). AUC of CRP was not significant and therefore not included in the figure

A NLR value of 6.2 (9.03), was proposed as a threshold with both sensitivity and specificity > 90% for bacterial infection (fungal infection) [20, 21]. To evaluate the performance of inflammation markers in lung and liver transplant recipients, ROC analysis was performed. NLR, neutrophils, lymphocytes, and IL-6 were the inflammation markers that demonstrated usefulness in terms of sensitivity and specificity in the detection of bacterial and fungal specimens (Fig. 2). The largest AUC was observed for the NLR (0.631; *p* < 0.001). The optimal NLR threshold for infection, based on the Youden index, was 12.0 (sensitivity: 74.0%, specificity: 50.7%, J = 0.246,). In ICU patients who underwent liver or lung transplantation, overall sensitivity for the detection of microbial samples with an NLR cutoff of 6.2 was 90.5% with a specificity of 21.8% (J = 0.122). The AUC for IL-6 was 0.579 (*p* < 0.001). A cutoff of 22.6 pg/mL (J = 0.147) showed the best combination of sensitivity (50.4%) and specificity (64.4%). The AUCs for neutrophils and lymphocytes were 0.550 (*p* = 0.001) and 0.608 (*p* < 0.001), and cutoffs were determined at 7.745 G/L (J = 0.121, sensitivity: 64.5%, specificity: 47.6%) and 0.505 G/L (J = 0.202, sensitivity: 57.0%, specificity: 63.2%).

### Detection frequency of positive bacterial and fungal samples is increased with the addition of leukocytes

**Fig. 3:**
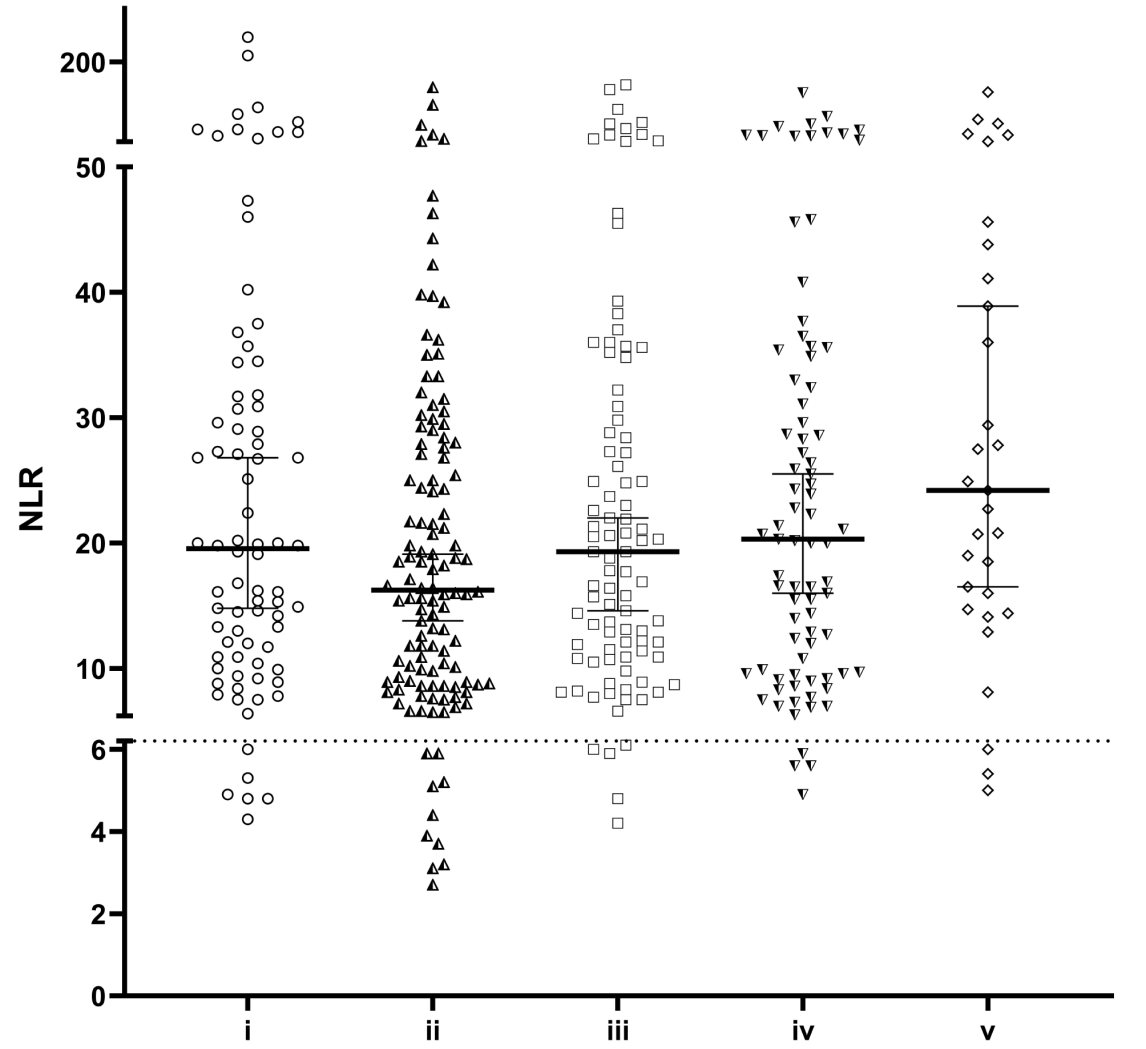
Frequency of elevated NLR as an inflammation marker is increased, if leukocytes are elevated. The NLR was highest in patients with the combination of Gram-positive/Gram-negative bacteria (v) (24.2; [16.5, 38.9]), but lowest in patients infected with Gram-negative bacteria only (ii) (16.3; [13.8, 19.1]. The dotted line indicates the proposed threshold for NLR (6.2) for infection. (i: Gram-positive; ii: Gram-negative; iii: fungus; iv: combination of bacteria and fungus; v: combination of Gram-positive and Gram-negative)

In order to increase the detection frequency of bacterial and fungal samples a two-step analysis of inflammation markers was evaluated. If leukocytes were increased (≥ 9.8 G/L), the NLR was tested against the threshold of 6.2. The NLR was highest in patients tested positive for a combination of Gram-positive/Gram-negative bacteria (v) (24.2; [16.5, 38.9]), and lowest in patients with Gram-negative bacteria only (ii) (16.3; [13.8, 26.8]) (Fig. 3).

NLR values > 6.2 were more often associated with positive microbiology results, if leukocytes were added to the decision process. The frequency could be increased for all specimen categories (i-v) from 78.2 to 93.1% The frequency of NLR values ≥ 6.2, if Gram-positive bacteria (i) were detected, was 92.3% (+ 17.5%); it was 91.8% (+ 21.0%) for Gram-negative bacteria (ii), 94.6% (+ 8.6%) for fungi (iii), 95.1% (+ 7.9%) for both bacteria and fungi (iv) and 93.1% (+ 14.9%) for both Gram-positive and Gram-negative bacteria (v).

The initial screening for elevated CRP (or IL-6) did not lead to significant changes in the detection frequency for microbial specimens for NLR values > 6.2. The frequency of NLR values ≥ 6.2 for the detection of Gram-positive bacteria was 73.8% [-1.0%] (76.8% [+ 2.0%]); it was 69.7% [-1.1%] (70.8% [± 0.0%]) for Gram-negative bacteria, 85.2% [-0.8%] (85.7% [-0.3%]) for fungi, 87.4% [+ 0.3%] (87.6% [+ 0.5%]) for both bacteria and fungi and 86.0% [± 0.0%] (81.1% [-5.1%]) for both Gram-positive and Gram-negative bacteria.

### The NLR is independent of organ type or patient gender

**Fig. 4:**
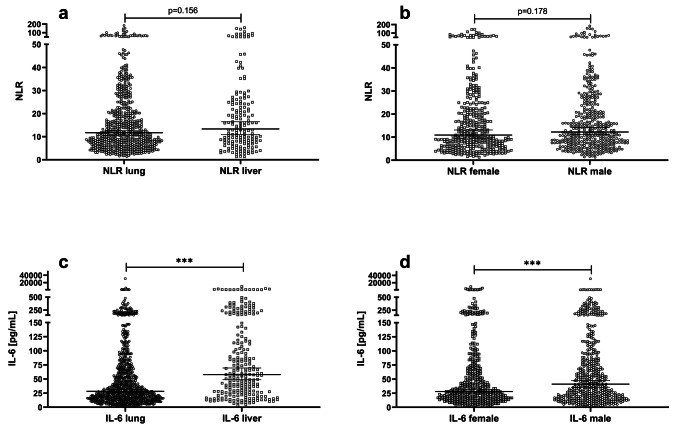
Concentration of the NLR is independent of type of organ transplant and patients gender in contrast to Interleukin-6. The NLR values were similar in patients who are (**a**) lung or liver recipients (11.7 [10.6, 12.6] vs. 13.4 [10.9, 16.5]; *p* = 0.156) and (**b**) female or male (10.9 [9.6, 13.1] vs. 12.2 [11.1, 14.2]; *p* = 0.178). IL-6 values were higher in (**c**) liver recipients (57.9 pg/mL [48.8, 69.4] vs. 28.2 pg/mL [26.0, 31.7]; *p* < 0.001) and in (**d**) male patients (41.1 pg/mL [35.6, 47.4] vs. 28.1 pg/mL [25.4, 32.0]; *p* < 0.001)

To investigate whether the NLR is a stable and reliable marker, the covariables patient gender and type of organ transplant were analysed separately. NLR values were similar in patients who received lung or liver transplant (11.7 [10.6, 12.6] vs. 13.4 [10.9, 16.5]; *p* = 0.156) (Fig. 4.a). Stratification by gender showed no difference in NLR values (women: 10.9 [9.6, 13.1] vs. men: 12.2 [11.1, 14.2]]; *p* = 0.178) (Fig. 4.b). In contrast, IL-6 concentration differed significantly depending on the type of organ (liver: 57.9 pg/mL [48.8, 69.4] vs. lung: 28.2 pg/mL [26.0, 31.7]; *p* < 0.001) (Fig. 4.c) and between female and male patients (28.1 pg/mL [25.4, 32.0] vs. 41.1 pg/mL [35.6, 47.4]; *p* < 0.001) (Fig. 4d). In addition, the NLR showed only a moderate to no correlation with other inflammation markers ((A) WBC vs. NLR: *r* = 0.552; *p* < 0.001, B) CRP vs. NLR: *r* = 0.240; *p* < 0.001, C) IL-6 vs. NLR: *r* = 0.173; *p* < 0.001, D) PCT vs. NLR: *r* = 0.460; *p* = 0.012)) (Additional Fig. 2).

## Discussion

The objective of this study was to investigate the effectiveness of the NLR as a refined cost-effective method of detecting bacterial and fungal specimens in lung and liver transplant patients. The NLR values differed between different types of infection-causing microbes with overall observed frequency for an NLR value > 6.2 between 70 and almost 90%. If only patients with elevated WBC were considered, the frequency of elevated NLR values > 6.2 was increased to > 90% for all types of infection-causing microbes. The NLR demonstrated to be a better marker for positive bacterial and fungal microbiology findings than IL-6, CRP or leukocytes, according to results of an ROC analysis. All commonly used inflammation markers demonstrated a low specificity. Analyses of the covariables patient gender and type of organ transplant revealed that the NLR can be used as an independent indicator in contrast to IL-6, the concentrations of which differed significantly between male and female patients and between lung and liver transplanted patients.

Combinations of different types of microbes led to higher NLRs, probably due to more pronounced activation of the immune system. Neutrophils proliferate earlier, hence the neutrophil fraction of the WBCs increases; later lymphocytes start to proliferate [22]. However, cellular changes also occur after stressful events like surgery or trauma [23]. In addition, corticosteroids (all patients received corticosteroids) increase the neutrophil fraction of the WBC count [24]. Interestingly, among patients who had positive microbiology results just once (Additional Fig. 3), there were no differences in NLR values between types of microorganisms. However, the highest NLR values were also present in patients tested positive for the combination of Gram-positive and Gram-negative bacteria (v) and lowest in Gram-negative bacteria (ii).

The sensitivity of a biomarker is crucial to detect an infection, but a perfect marker requires high specificity to minimize false positive results. ROC analysis, which considers both sensitivity and specificity, demonstrated that all infection markers are far from being perfect. Nonspecific elevation of biomarkers in the setting of trauma or surgery might be a reason for this [25]. IL-6, a pro-inflammatory cytokine, has demonstrated high sensitivity for inflammation. It is released early in the inflammation cascade and has been used as an early marker of sepsis [26], but it is also increased after surgical procedures and trauma [25]. This might be why the IL-6 concentrations were higher in this subset of patients and why its use in the detection of positive microbiology results is limited.

Another important feature of a perfect infection marker is universal use, independent and robust against cofactors. There were no differences in the NLR with regard to transplanted organ type, but IL-6 concentrations differed significantly, with higher circulating IL-6 concentrations in liver transplant patients. IL-6 concentrations are increased after liver resection, and in phases of liver regeneration [27, 28], which can be assumed to be similar in patients after liver transplantation. In these cases, the benefits of measuring IL-6 and CRP to detect bacterial and fungal specimens are limited (synthesis of CRP is induced by IL-6). NLR values were similar between male and female patients, whereas IL-6 concentrations were higher in men. In general, men seem to be more susceptible to infection with higher levels of inflammation markers [29]. This may be attributed due to the different sex hormones between men and women [30].

Holub et al. reported that an NLR cutoff of > 6.2 has both a sensitivity and a specificity of > 90% for the detection of bacterial infection [20]. In this study we observed much lower sensitivity and specificity at an almost doubled cutoff (12.0) for the NLR. For immunosuppressed patients, who are in the convalescence period after solid organ transplantation, the detection of bacterial and fungal specimens, and more important the detection of an infection, might be more important than specificity. The much lower specificity in comparison to Holub et al. might be due to the different subset of patients, as the patients here in this study were all postoperative patients after a life-changing procedure, under immunosuppression with a variety of bacterial and fungal specimens.

As described, the patients in this study showed much higher concentrations/levels of inflammatory markers, hence the reference values were not sufficient to detect bacterial and fungal samples for all inflammatory markers, and a more resilient marker might help in the decision process. Leukocyte count is not considered to be a good parameter for the detection of infection/sepsis. Here, the addition of leukocytes markedly increased the frequency of NLR values > 6.2 in patients with multiple positive microbiology results for bacteria or fungus.

The study had some limitations. The retrospective design did not allow us to draw conclusions about patient outcomes. The process of deciding to begin antibiotic therapy involves a consideration of the kinetics of more than one marker and includes clinical aspects of the patient’s current status. Apart from one study that demonstrated that the red blood cell distribution width and the NLR were elevated in correlation with infection in patients who underwent liver transplantation [31], this is the largest real-world study to evaluate the NLR based on blood samples obtained from patients who underwent lung or liver transplantation. Two general problems in all studies involving microbiology testing are the sensitivity of the microbiology tests and the possibility of sampling errors. To address these issues, we focused on patients who were tested positive multiple times for microbiology specimens.

## Conclusion

In summary, the infection monitoring of lung and liver transplanted patients is challenging due to increased inflammation marker postoperatively. The NLR is a cost-effective and widely available inflammation marker, with similar or more value for detection of bacterial and fungal specimens compared to other inflammation markers in lung- or liver transplanted patients.

## Electronic supplementary material

Below is the link to the electronic supplementary material.

Supplementary Material 1

Supplementary Material 2

Supplementary Material 3

Supplementary Material 4

## Data Availability

The data that support the findings of this study are available on request from the corresponding author SBW.

## Abbreviations

AUC: Area under curve
CRP: C-reactive protein
ICU: Intensive care unit
IL-6: Interleukin-6
NLR: Neutrophil-to-lymphocyte ratio
PCT: Procalcitonin
ROC: Receiver operating characteristic
SD: Standard deviation
WBC: White blood cell
