# Supplementary material for: Importance of the neutrophil-to-lymphocyte ratio as a marker for microbiological specimens in critically ill patients after liver or lung transplantation
Source: Infection. 2024 Nov 25;53(2):573–82. doi: 10.1007/s15010-024-02398-4 (PMC11971184; doi:10.1007/s15010-024-02398-4)
Supplement: Supplementary file 1 — Supplementary Material 1 [file 15010_2024_2398_MOESM1_ESM.pdf]

Figure 1	i	ii	iii	iv	v
Number of values	163	329	143	132	50
Minimum	1,3	1,3	2,6	3,1	2,2
25% Percentile	6,1	5,55	8,1	8,45	9,475
Median	11,1	9,1	13,5	15,05	18,4
75% Percentile	20,8	17,8	26,1	28,03	33,23
Maximum	246,5	152,1	184,3	142,2	143,3
Range	245,2	150,8	181,7	139,1	141,1
95% CI of median					
Actual confidence level	95,86%	95,30%	95,56%	95,51%	96,72%
Lower confidence limit	9,4	8,5	10,9	12	14,1
Upper confidence limit	13,9	10,6	17	18,4	24,2
Mean	20,02	14,72	22,71	22,58	27,53
Std. Deviation	29,83	17,26	27,47	21,86	28,47
Std. Error of Mean	2,336	0,9515	2,297	1,903	4,027

Figure 3	i	ii	iii	iv	v
Number of values	80	122	93	81	31
Minimum	4,3	2,7	4,2	4,9	5
25% Percentile	10,9	8,875	11,15	9,65	14,7
Median	19,55	16,25	19,3	20,3	24,2
75% Percentile	31,78	27,93	31,55	35,15	43,8
Maximum	246,5	152,1	157,1	142,2	143,3
Range	242,2	149,4	152,9	137,3	138,3
95% CI of median					
Actual confidence level	96,70%	96,31%	96,25%	95,52%	97,06%

Lower confidence limit	14,8	13,8	14,6	16	16,5
Upper confidence limit	26,8	19,1	22	25,5	38,9
Mean	30,81	21,38	26,72	27,47	33,95
Std. Deviation	39,04	20,12	26,87	24,24	29,9
Std. Error of Mean	4,365	1,821	2,786	2,693	5,371

**Figure 4**	**IL-6 male**	**IL-6 female**	**NLR male**	**NLR female**
Number of values	526	454	414	358
Minimum	1,5	2,7	1,3	1,3
25% Percentile	17,8	15,6	7,5	6,1
Median	41,1	28,1	12,2	10,9
75% Percentile	92,7	66,5	23	22,5
Maximum	30748	8834	212	247
Range	30747	8831	211	245
95% CI of median				
Actual confidence level	95,00%	95,70%	95,60%	96,10%
Lower confidence limit	35,6	25,4	11,1	9,6
Upper confidence limit	47,4	32	14,2	13,1
Mean	149	115	19,6	19,3
Std. Deviation	1346	542	23,8	24
Std. Error of Mean	58,7	25,4	1,17	1,27

	IL-6Lung	IL-6Liver	NLRLung	NLRLiver
Number of values	741	239	610	162
Minimum	1,5	2,8	1,4	1,3
25% Percentile	15	26,4	6,6	7,5

Median	28,2	57,9	11,7	13,4
75% Percentile	67,3	143	21,6	24,9
Maximum	30748	8834	247	157
Range	30747	8831	245	156
95% CI of median				
Actual confidence level	95.3%	96,20%	95,30%	95,10%
Lower confidence limit	26	48,8	10,6	10,9
Upper confidence limit	31,7	69,4	12,6	16,5
Mean	111	203	18,9	21,8
Std. Deviation	1149	669	23,3	25,7
Std. Error of Mean	42,2	43,3	0,945	2,02

**Table 1**	**Leukocytes**	**CRP**	**IL-6**	**PCT**	**NLR**	**neutrophils**	**lymphocytes**
Number of values	5067	4989	4854	364	3743	5067	3743
Minimum	0,51	0,1	1,5	0,1	0,8	0,1	0,02
25% Percentile	6,33	1,7	14,5	0,5	7,5	4,7	0,36
Median	10,2	4,1	30,5	1,8	13,8	8,11	0,58
75% Percentile	15,2	8,15	70,1	5,58	25	13	0,93
Maximum	90,4	48,3	384000	117	433	79,4	4,01
Range	89,9	48,2	383999	117	432	79,3	3,99
95% CI of median							
Actual confidence level	95,00%	95,00%	95,00%	95,90%	95,00%	95,00%	95,00%
Lower confidence limit	9,95	3,9	29	1,4	13,3	7,94	0,56
Upper confidence limit	10,4	4,3	31,7	2,4	14,3	8,29	0,6

Mean	12,1	5,78	259	8,84	20,1	9,9	0,705
Std. Deviation	8,83	5,58	7067	19,9	22,7	7,76	0,496
Std. Error of Mean	0,124	0,0789	101	1,04	0,372	0,109	0,00811
