# Supplementary figures and images for: Importance of the neutrophil-to-lymphocyte ratio as a marker for microbiological specimens in critically ill patients after liver or lung transplantation
Source: Infection. 2024 Nov 25;53(2):573–82. doi: 10.1007/s15010-024-02398-4 (PMC11971184; doi:10.1007/s15010-024-02398-4)

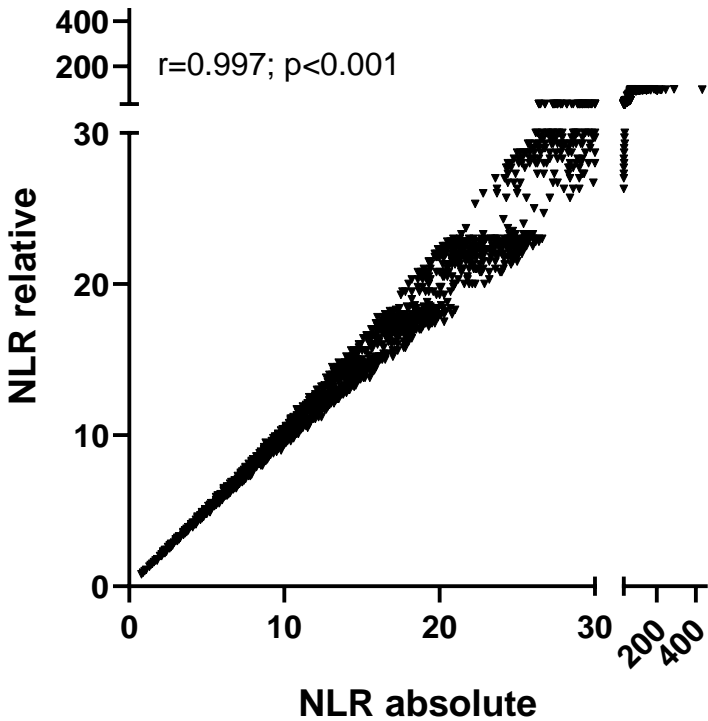

Supplement: Supplementary file 2 — Supplementary Material 2 [file 15010_2024_2398_MOESM2_ESM.pdf]

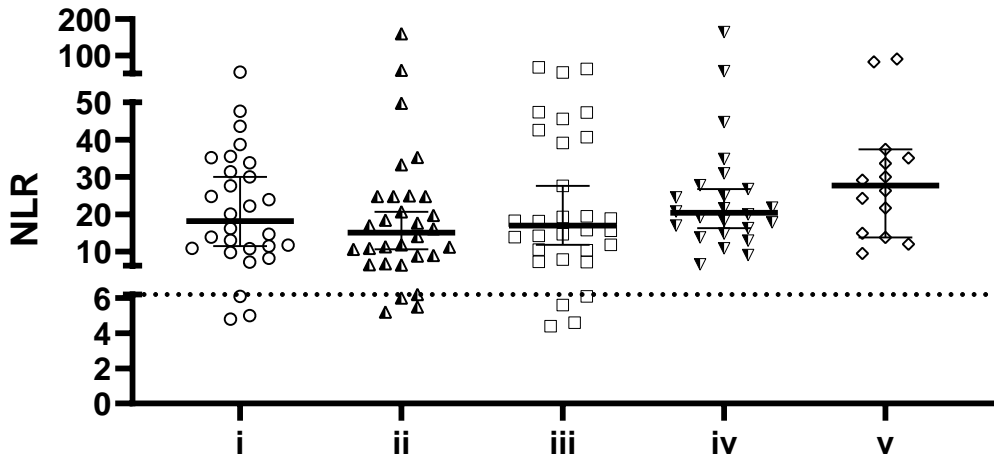

Supplement: Supplementary file 3 — Supplementary Material 3 [file 15010_2024_2398_MOESM3_ESM.pdf]

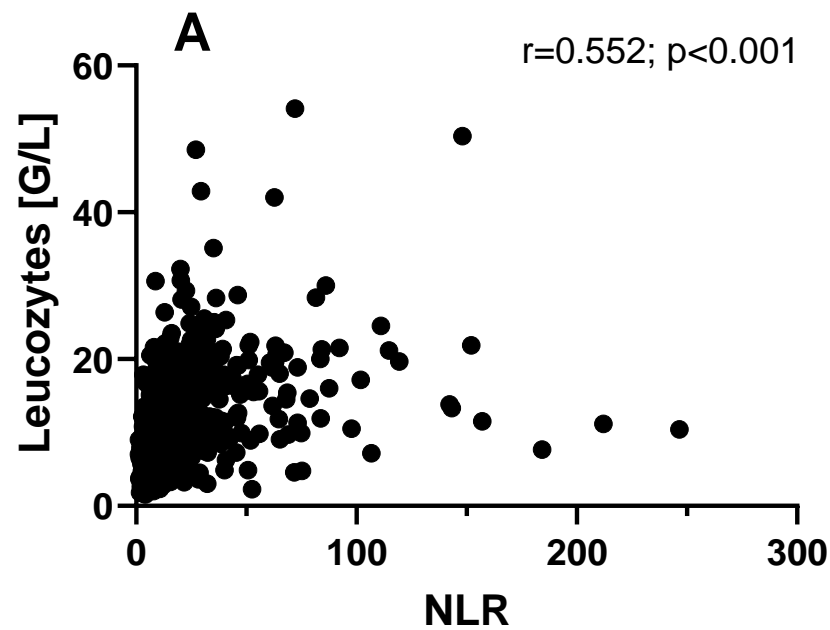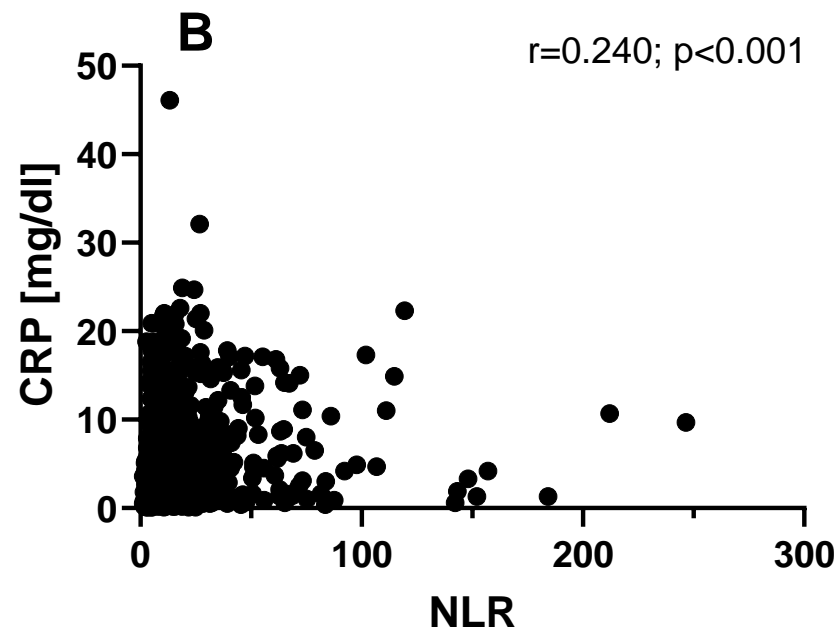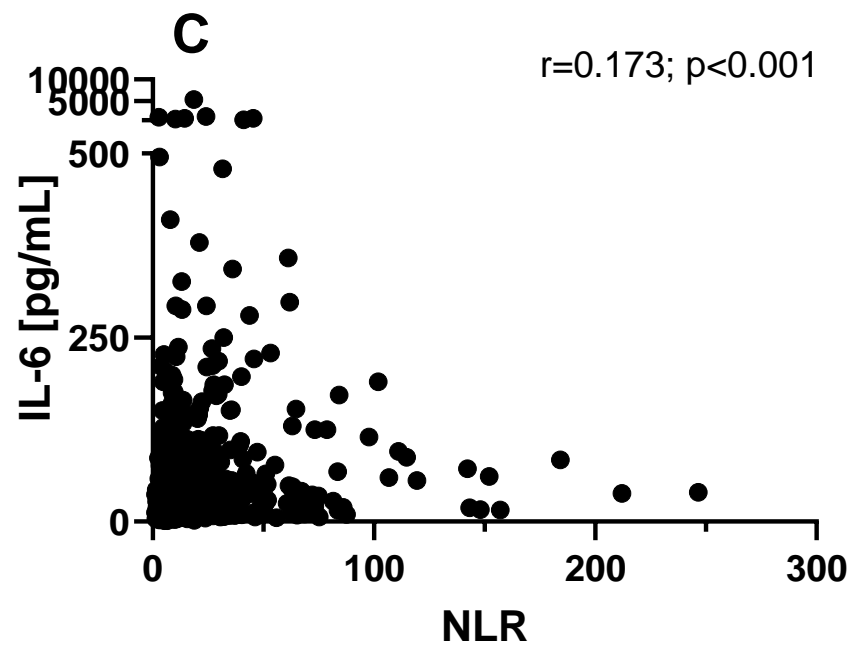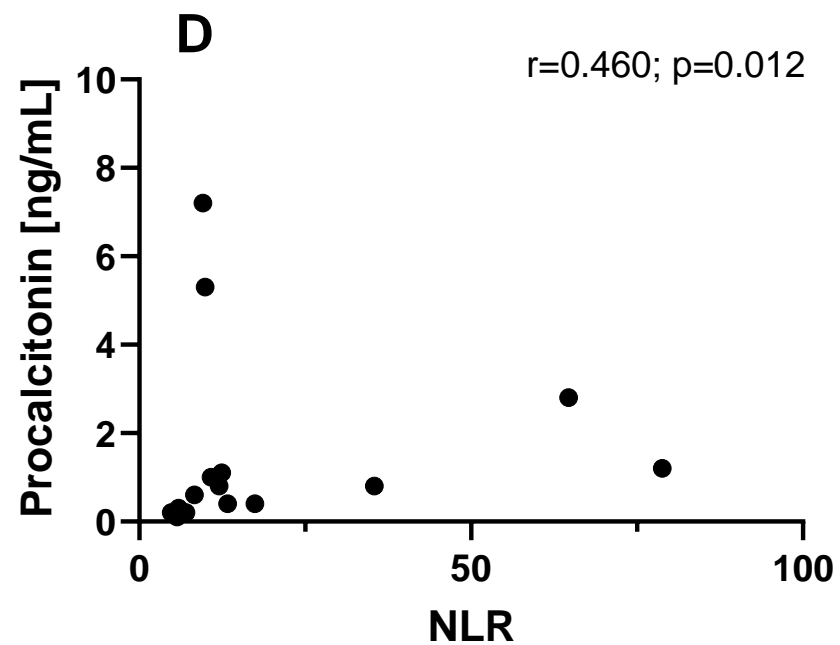

Supplement: Supplementary file 4 — Supplementary Material 4 [file 15010_2024_2398_MOESM4_ESM.pdf]
